# Pennsylvania College, Ninth below Locust Street

**Published:** 1853-04

**Authors:** W. H. Gobrecht


### CLINICAL REPORTS.

*Pennsylvania College, Ninth below Locust street. Service of* Professor GILBERT.

Reported by W. H. GOBRECHT, M. D.

*Feb. 9th.—Abscess.*—Thomas C——, (Case LXXVII.) The abscess is filling up, and as far as the injury is concerned, the patient is improving. The case, however, is complicated with slight anasarca in a strumous diathesis; for which is ordered:

℞. Pil. Hydrargyri No. ii.

To be followed by:

℞. Potassæ bi-tartrat. ℥iij.
Pulv. Jalapæ. ℈ij.
M. S. Take a teaspoonful every three hours until purgation results.

Case LXXXIII.—*Inverted toe-nail,* in Rachel B——, aged 15. Ingrowing nail occurs more frequently in the foot than in the hand, the great toe being oftenest affected. It is caused by the pressure of tight boots or shoes upon the soft parts of the toes, forcing them up against the edges of the nail, thus burying it in their substance. The pressure thus produced by the edge or edges of the nail, causes inflammation and ulceration, and finally painful granulations arise, which overlie it, besides being impinged on by its edge, giving rise to so much pain that locomotion can not be performed. Moreover, these ulcerations may assume a cancerous character, if long neglected.

In treating inverted toe-nail, loose shoes should be ordered, and if the case be mild it may be cured, or at least much palliated, by gently stuffing cotton beneath the sharp edge of the nail to relieve its pressure, and applying caustic to the exuberant granulations. In bad cases, however, we can only cure by taking either the affected side or the whole of the nail away.

The patient presented had inversion of both lateral edges of the left great toe-nail, with ulceration and exuberant granulations about them.

It was removed according to the method of Dupuytren, that is, by introducing one blade of the sharp pointed scissors beneath the anterior edge of the nail at its centre, keeping close to it and passing down to the matrix. The blades being brought together and the nail divided, each half was wrenched from its bed by strong forceps. The ulcer remaining was dressed with Simple Cerate. This very painful operation was performed during partial Anæsthesia, induced by the inhalation of a mixture of Chloroform and Ether.

*Double Cataract.*—Margaret G——, (Case LXXII.) This patient is still improving. She sees faces but cannot distinguish them well. Pupil dilated in the presence of class with aqueous solution of Atropia.

16th. As this patient is anxious to return to her home in the country, it is thought proper to furnish her with a cataract glass, which is much more convex than those usually employed as spectacles, and is intended as a substitute for the absorbed lens. A number of such were presented, from which that best fitted to the peculiarities of her eye was selected, and by means of which she was enabled to see quite distinctly. She was directed not to use this for six or seven months to come, to avoid irritation and inflammation, or other changes of the retina, from the unaccustomed concentration of light upon its surface, which might result in the production of amaurosis.

Case LXXXIV. *Hare lip with cleft palate.*—Rebecca ——, aged 22. In this case very great deformity exists; the lip is not only deficient, but the cleft extends through the left superior maxillary and palate bones, whilst the aveolar processes and several teeth project at its anterior extremity, giving the patient an exceedingly unpleasant appearance, and materially affecting her powers of eating and speaking.

Besides the very great congenital deficiency of material to work with here, it is stated that an unsuccessful operation for relief had been previously performed; large cicatrices of a firm, fibrous character resulting.

The fræna existing on both sides of the fissure were detached, and the left side of the lip and ala of the nose freely dissected from the superior maxilla; a small wooden spatula was then placed successively beneath each margin of the cleft in the soft parts, upon which the fibrous edges were fully cut away by a sharp scalpel, the incisions being made concave to the fissure to ensure a horizontal margin to the new lip, which was then formed by the apposition of the cut edges by means of two large gold needles in the substance of the lip, and one smaller, just at its edge, the twisted suture being formed about them.

A broad adhesive plaster was then applied to each cheek as far back as the ear; each anterior extremity extending to the oral angle of its own side, was doubled upon itself, (thus forming a thick edge,) and connected with its fellow of the opposite side by ligatures passing between them; these ligatures being drawn tightly, the tension of the buccinator and other muscles of the cheeks was effectually controlled.

16th. The small needle was removed on the third, and both the largest at the end of the fourth, whilst the threads adhered until the fifth day.

To-day, on completely redressing, adhesion is found perfect in nearly every part, though granulation must occur at some points. Isinglass plaster was applied directly over the wound to defend the new material from the action of foreign matters; adhesive strips, similar to those previously employed, were used in a like manner; whilst pressure of the connecting threads upon the lip was prevented by placing a roll of lint beneath the anterior extremity of each strip.

*Feb. 12th. Spinal Irritation.*—Robert C——, (Case LXVII.) Has had no spasm since his last appearance here.

Whenever an extra veratria pill is taken, the tingling sensation, indicative of its action, results. The patient is evidently improving. The veratria is discontinued, to be resumed if necessary.

Case LXXXV. *Lumbago.*—Hugh L——, aged 21, boatman, presented himself as a surgical patient, but is discovered to have lumbago only, for which is prescribed:

| | | |
|---|---|---|
| ℞ | Potassii Iodidi, | ʒi. |
| | Vini Colchici Sem. | f℥i. |
| | Aquæ Menthæ virid. | f℥ii. |

M. S. A tea spoonful thrice daily, ceasing when it purges.

*Porrigo.*—Christiana S——, (Case LXXVII.) Improving under treatment.

Case LXXXVI. *Porrigo.*—Francis S——, aged 10. The disease in this case appeared at the eleventh month, just after vaccination, the mother attributing it to the use of improper virus in the operation; this is, however, doubtless an error. The affection has continued up to this time, and during the course of it, ophthalmia tarsi, which is a chronic inflammation of the edges of the lids, extending into the Meibomean follicles, from which a viscid abnormal secretion results, has arisen. The mother states that every variety of treatment has been employed, with low diet. There is prescribed free exercise in the open air, warm salt bath, followed by frictions with a coarse towel, and full diet; also,

℞. Hydrarg. oxid. rubr. gr. x.
Cerat. simp. ℥i.
M. ft. ung.
S. To be applied to the edges of the lids nightly.

Case LXXXVII. *Supposed polypus nasi.*—Mr. L——, aged 41, has been troubled for five or six years with a gradually increasing stoppage of the left nostril, attended by an alteration of the voice, and a polypus apparently existed upon the septum narium on the left side, an unusual spot for its development. On the application of the forceps, however, enlargement of the mucous membrane and periosteum were found to exist, but no polypi.

Case LXXXVIII. *Strabismus.*—Caroline F——, aged 6, has convergent strabismus of the right eye. It was stated that, in cases of such an age, surgical attempts at relief were perhaps of doubtful propriety, inasmuch as although the patient would be perfectly willing to enter into the operation, the subsequent struggles might be very embarrassing. The anxiety, however, of the parents for its cure, led the Surgeon to the performance of the operation, which was done in the usual manner, and with entire success, although, as expected, the struggles of the child were exceedingly annoying.

Case LXXXIX. *Inflammation of the Hand.*—Martha K——, aged 20, being affected with tetter, was recommended by her friends to apply corrosive sublimate made into an ointment with butter, which she did on the 8th inst. The quantity employed was large, and its application was followed by intense pain, swelling, redness and loss of sleep from the irritation set up, which has lasted, although now abating, to the present time.

The case is shown as an example of the results following the injudicious appliance of remedial means on popular recommendation.

A poultice was ordered as the only treatment necessary.

*Feb.* 16*th.* (Case LXXV.) *Amaurosis.*—Albion D. S——. Reported as recovering vision rapidly.

19th.—Can now see with both eyes, though least with the right. The Iritis which existed in it is yielding rapidly and is almost subdued. The calomel powders are continued with occasional purgation.

The patient is informed that he must change his occupation to one requiring less stooping, since this aids in the production of congestion of the head, caused also by holding the breath during the exertion of hauling upon ropes, which he must pursue as a boatman and oyster dredger.

Case XC.—*Congenital double Inguinal Hernia*, occurring in James B——, aged 5 months.

Remarks upon the pathology of this disease were given, and a double truss applied, which is to be worn for three or four months.

23d.—Truss removed and re-applied; child doing very well.

Case XCI. *Subacute Conjunctivitis.*—John H——, aged 33, was seized with inflammation of the eyes, about two weeks since, and this has now assumed the subacute form. The conjunctivæ alone are involved. Besides local treatment, his digestive organs require correction.

His tongue being enlarged, with a white coat upon it, appetite poor, and the bowels somewhat constipated, ordered

℞. Mass. Pil. Hydrarg. ℈j.

Pulv. Aloes ℈ss.

Pulv. Rhei ℈ss.

M. ft. in pil., No. X.

S. Three to be taken at bed time. The others as required. If the three pills do not move the bowels, a seidlitz powder is to be taken the next morning.

As a local application to the eye,

℞. Zinci Chloridi gr. ss.
Aq. Rosæ f℥j.
M.

S. Apply to conjunctivæ once a day.

Case XCII. *Parotitis.*—Wm. S——, printer, aged 22. The swelling of the left parotid gland commenced on the 13th inst., since which time it has been increasing in size, being hard and painful. He is directed to apply linim. ammoniæ to the part, to be purged freely with magnes. sulphat., and to be restricted in diet.

Case XCIII. *Epiphora.*—James P——, aged 44. This results from obstruction in the nasal duct. Creasote is painted over the lachrymal sac and the course of the duct as far as practicable.

*Feb. 23d.* Case XCIV. *Fracture at and Luxation of right elbow joint.*—Thos. McG——, aged 7. The injury was sustained by this patient 23 days since, and 16 days after its occurrence he appeared for treatment, when the right fore-arm was found rigidly extended; the coronoid process was fractured and drawn up on the arm anteriorly, whilst the olecranon was drawn upward posteriorly, the ulna of course following it. The fore-arm was first drawn downward by means of extension and counter-extension, and then flexed; the parts being thus properly adjusted were retained in position by a splint with a movable angle, controlled by a Stromeyer's screw, at the elbow, applied on the anterior aspect of the limb.

The following sedative lotion was ordered to be applied to the joint by wetted cloths.

℞. Plumbi Acetat. ʒij.

Morphiæ Acetat. gr. x.

Aquæ Oj.

M. ft. lot.

The angle of the splint, after some little time has elapsed, to be changed frequently, but gently and gradually, and if anchylosis seems inevitable, it is to be allowed in the flexed position only, since the arm is not then useless, as it is if rigidly extended.

In concluding this series of reports, the history of a case presented to the class on Dec. 11th, 1852, will be given, as omitted at that time.

*Dec.* 11*th.* CASE XCV. *Hydrocele of the Neck.*—Mrs. M——, aged 50, is presented, with a large tumor, of 18 years' growth, involving the entire anterior and lower portion of the neck; on examination this is found to project downward behind the sternum, to either side beneath the sterno-cleido mastoidei muscles, and upward as high as the upper edge of the thyroid cartilage. It would thus involve important structures, and might arise from several causes; our diagnosis must therefore be accurate. It is found to have pulsation and thrill, but this doubtless arises from overlying the carotid arteries, inasmuch as when the tumor is pressed away, the vessel of the relieved side can be distinctly felt, whilst no thrill and but very indistinct pulsation is communicated to that portion of the tumor. This also occurs when the other side is examined, besides which we cannot either diminish the size or empty it by pressure; it is therefore not an aneurism. It is not bronchocele, for its history tells us that it commenced in the median line and in the lower part of the neck. Now, had it been bronchocele, the lobes of the thyroid body would in all probability have been first affected, and this higher up than the present disease commenced; besides which, goitre is usually hard, whilst this is soft, especially upon the left side, and fluctuation is very evident upon palpation. It is, therefore, doubtless filled with fluid, (which so readily transmits the impulse of the vessels to the fingers,) and is a hydrocele;—a cyst,—whose contents are increasing, since they are secreted more rapidly than absorption is performed. Such tumors are generally located beneath the platysma myoides m., and upon the larynx. The removal of this tumor or the diminution of its size is important, since, being of 18 years' standing, it is large, and now increases rapidly, producing great deformity, whilst it presses on the trachea, keeping up a chronic congestion of the air passages, rendering breathing difficult, and thus, especially during the summer, rendering the slightest forms of labor very difficult for her performance.

On account of the size of this tumor, and the number of important parts involved, the operation here indicated would be exploratory in its character, the fluid would then be withdrawn, and if the sac was in a proper state, iodine injection would be resorted to, with the intention of producing adhesion between its walls.

This patient, as previously stated, will present herself in future for operation.

